# Genome-Wide Identification and Expression Analysis of Potential Antiviral Tripartite Motif Proteins (TRIMs) in Grass Carp (*Ctenopharyngodon idella*)

**DOI:** 10.3390/biology10121252

**Published:** 2021-12-01

**Authors:** Beibei Qin, Tiaoyi Xiao, Chunhua Ding, Yadong Deng, Zhao Lv, Jianming Su

**Affiliations:** Hunan Engineering Technology Research Center of Featured Aquatic Resources Utilization, Hunan Agricultural University, Changsha 410128, China; beibeiqin@stu.hunau.edu.cn (B.Q.); tiaoyixiao@hunau.edu.cn (T.X.); chunhuading@stu.hunau.edu.cn (C.D.); ya-dong.deng@stu.hunau.edu.cn (Y.D.)

**Keywords:** tripartite motif proteins, B30.2 domain, antiviral immunity, *Ctenopharyngodon idella*, grass carp reovirus

## Abstract

**Simple Summary:**

Grass carp, *Ctenopharyngodon idellus*, is an important freshwater cultured teleost in China, and its annual production has reached 5,533,083 tons. However, its aquaculture is severely restricted by hemorrhagic disease caused by the grass carp reovirus (GCRV). For the better control of grass carp hemorrhagic disease, the breeding of resistant grass carp strains based on antiviral immune molecule markers is a potential solution. However, the molecular basis of grass carp’s resistance to GCRV infection remains largely unknown, greatly limiting the breeding of grass carp resistant to hemorrhagic disease. Given the importance of tripartite motif proteins (TRIMs) in animal antiviral immunity, we used the Hidden Markov Model Biological Sequence Analysis software (HMMER) and SMART to identify TRIMs in the grass carp genome and analyze their gene loci, as well as structural and evolutionary features. We also tried to uncover antiviral TRIMs and their mediated immune processes based on two sets of transcriptomes during GCRV infection in grass carp. This study provides information for the understanding of TRIMs and antiviral immunity in grass carp.

**Abstract:**

Tripartite motif proteins (TRIMs), especially B30.2 domain-containing TRIMs (TRIMs-B30.2), are increasingly well known for their antiviral immune functions in mammals, while antiviral TRIMs are far from being identified in teleosts. In the present study, we identified a total of 42 *Ci*TRIMs from the genome of grass carp, *Ctenopharyngodon idella*, an important cultured teleost in China, based on hmmsearch and SMART analysis. Among these *Ci*TRIMs, the gene loci of 37 *Ci*TRIMs were located on different chromosomes and shared gene collinearities with homologous counterparts from human and zebrafish genomes. They possessed intact conserved RBCC or RB domain assemblies at their N-termini and eight different domains, including the B30.2 domain, at their C-termini. A total of 19 TRIMs-B30.2 were identified, and most of them were clustered into a large branch of *Ci*TRIMs in the dendrogram. Tissue expression analysis showed that 42 *Ci*TRIMs were universally expressed in various grass carp tissues. A total of 11 significantly differentially expressed *Ci*TRIMs were found in two sets of grass carp transcriptomes during grass carp reovirus (GCRV) infection. Three of them, including *Ci*btr40, *Ci*TRIM103 and *Ci*TRIM109, which all belonged to TRIMs-B30.2, were associated with the type I interferon response during GCRV infection by weighted network co-expression and gene expression trend analyses, suggesting their involvement in antiviral immunity. These findings may offer useful information for understanding the structure, evolution, and function of TRIMs in teleosts and provide potential antiviral immune molecule markers for grass carp.

## 1. Introduction

Tripartite motif proteins (TRIMs) are generally characterized by three domains at the N-terminus, including a RING finger domain, one or two B-box domains and a coiled-coil domain, and are also known as the RING finger/B-box/coiled-coil (RBCC) domain-containing proteins [1,2]. The B-box domains represent a very ancient domain family that can be traced back to a common ancestor in protozoa, metazoa and even plants [3,4]. Meanwhile, the complete RBCC domain assemblies have only been discovered in TRIM family proteins from metazoa, such as arthropods, teleosts, amphibians, birds and mammals [4,5,6,7]. It has recently been confirmed, in mammals and teleosts, that proteins only containing the RING finger domain and B-box domains (RB domain assemblies) also exert biological functions similar to those containing RBCC domain assemblies, which further expands the TRIM family of proteins [8,9]. Although the RBCC or RB domain assemblies appear to be conserved in animals, almost every species has more than one TRIM or a specific repertoire of TRIMs, all of which, together, constitute a large protein family with highly variable sequences [4,10]. These TRIM family proteins play multiple roles in animal tissue development [11], metabolism and autophagy [12], transcriptional regulation [13], tumor suppression [14] and viral restriction [15].

Most TRIM family proteins possess an additional distinct domain at the C-terminus, including at least 11 categories of domains such as the Plant Homeo Domain (PHD), meprin and TRAF homology domain (Math), bromodomain (BROMO) and B30.2 domain (constituted by the juxtaposition of a PRY and a SPRY domain, alternatively called the PRY/SPRY domain) [16]. The C-terminal domains often determine the specificity of the interactions of TRIMs with other proteins [12,17]. Hence, TRIM proteins’ RING-dependent E3 ubiquitin ligase activity is associated with the capacity to build multiprotein complexes though interactions with C-terminal domains. According to the categories of the C-terminal domains, the mammalian TRIM family proteins can be classified into nine main subsets, further extended to eleven subsets, according to Ozato’s nomenclature [15]. Among the identified C-terminal domains, B30.2 domains are the most frequent in TRIMs. In humans, 35 TRIMs containing the B30.2 domain (TRIMs-B30.2) have been found at the C-termini of 80 TRIM family proteins [16]. In addition, these TRIMs-B30.2 evolve significantly faster than other TRIMs based on the calculated ratio of the non-synonymous substitution rate (*Ka*) to the synonymous substitution rate (*Ks*) [4]. Recently, several studies have identified a cluster of TRIM-B30.2 genes flanking the human major histocompatibility complex (MHC) gene locus, a well-known immune gene [18]. Functional experiments have also demonstrated that TRIMs-B30.2 tend to be involved in host immune defense against viral infections in mammals. For example, human TRIM5a recognizes human immunodeficiency virus-1 via the B30.2 domain and inhibits viral replication by promoting the degradation of the viral outer capsids [19]. TRIM21 can bind to hepatitis B virus through the B30.2 domain and participate in the degradation of the viral DNA polymerase [20]. Evidence indicates that the B30.2 domain of TRIMs is critical for antiviral immunity in mammals.

In teleosts, TRIMs-B30.2 have undergone a massive expansion under positive selection pressure and duplicated to develop into three subfamilies: bloodthirsty-like (btr), hematopoietic lineage switch-5 (hltr) and fintrim (ftr) [10,21,22]. In the zebrafish genome, the TRIMs-B30.2 consist of 33 btrs, 43 hltrs and 88 ftrs [10]. The results of a molecular evolution analysis indicate that teleost hltrs and TRIM35 can be traced back to a common ancestor, while teleost btrs and TRIM39 share a common ancestor [10,21]. Mammalian TRIM35 and TRIM39 are well known for their immune-defense roles in viral infections [23,24]. In teleosts, a new btr gene has been identified in Atlantic cod, *Gadus morhua*, and detected in a poly I:C subtractive library [25]. In zebrafish, the btr subfamily TRIM gene btr20 shows high expression levels in immune tissues, including the intestines, gills, kidneys, and spleen [26]. Therefore, teleost btrs and hltrs are also believed to participate in antiviral immunity. To date, no common ancestor of ftrs has been found in the TRIM family proteins from other species, and ftrs are considered to be teleost-specific TRIMs [22]. Zebrafish ftr83 has been confirmed to regulate the expression of interferon (IFN) and IFN-stimulating genes and is involved in the immune defense against infectious hematopoietic necrosis virus, viral hemorrhagic septicemia virus (VHSV) and spring viremia of carp virus infection [27]. Scattered evidence suggests conserved roles for TRIMs-B30.2, including btrs, hltrs and ftrs, in antiviral immunity in teleosts. However, teleost antiviral TRIMs are far from being identified due to the gap in research on fish immunology and many TRIM family proteins. 

Transcriptome analyses, including weighted network co-expression analysis (WGCNA) and gene expression trend analysis, represent useful methods with which to survey host anti-infection immune molecules because they provide genome-wide profiles of gene expression [28,29,30]. In humans, a set of key immune genes, including IFN, interferon regulatory factor (IRF) 1, IRF 7, etc., interacting with human immunodeficiency virus (HIV)-1 have been defined by using WGCNA to construct gene co-expression networks based on transcriptome data from 52 patients [31]. Several antiviral immune genes, including laboratory of genetics and physiology 2 (LGP2), transforming growth factor-β-activated kinase 1 (TAK1) and zinc finger protein 36 (ZFP36), activated during encephalomyocarditis virus infection, have also been identified through gene expression trend analysis based on transcriptome data [32]. In teleosts, Ning et al. employed gene expression trend analysis combined with WGCNA to identify the differentially expressed gene clusters associated with anti-infection immune processes, including cytokine–cytokine receptor signaling, Toll-like receptor signaling and other immune-related pathways, from the transcriptomes of Japanese flounder (*Paralichthys olivacrus*) infected with *Vibrio anguillarum* [33].Recently, 10 hltrs associated with the type I IFN response significantly upregulated during VHSV infection were also identified as antiviral TRIMs by transcriptome analysis in rainbow trout [34].

Grass carp, *Ctenopharyngodon idellus*, is an important freshwater cultured teleost species in China, and its annual production has reached 5,533,083 tons [35]. However, the aquaculture of grass carp is severely restricted by grass carp hemorrhagic disease, which is caused by a double-stranded RNA virus known as the grass carp reovirus (GCRV) [36]. To better control grass carp hemorrhagic disease, it is urgent to investigate the molecular basis of grass carp’s ability to resist GCRV infection, and the breeding of resistant grass carp strains based on antiviral immune molecule markers is a potential solution [37]. However, the molecular basis of grass carp resistance to GCRV infection remains largely unknown, greatly limiting the breeding of grass carp resistant to hemorrhagic disease [38]. Therefore, the genome-wide identification of antiviral immune molecules could uncover the molecular basis of GCRV resistance in grass carp and contribute to disease resistance breeding. Given the importance of TRIMs, especially TRIMs-B30.2, in animal antiviral immunity, we used the Hidden Markov Model Biological Sequence Analysis software (HMMER) in the present study to screen TRIM family genes in the grass carp genome and identify TRIMs-B30.2 in line with their structural and evolutionary features. We also tried to identify potential antiviral TRIMs by analyzing the gene expression patterns during GCRV infection in two sets of transcriptome data in grass carp by using WGCNA and gene expression trend analysis. This study may not only find potential antiviral immune molecule markers for disease resistance breeding in grass carp, but also provide useful information for understanding the structure, evolution, and function of TRIMs in teleosts.

## 2. Materials and Methods

### 2.1. Identification of TRIMs in the Grass Carp Genome

The grass carp genome data were downloaded from the Grass Carp Genome Database (GCGD, http://bioinfo.ihb.ac.cn/gcgd/php/index.php, accessed on 20 November 2020) [39]. Putative TRIMs were first retrieved from the grass carp genome using the HMMER3.1 software with a multi-sequence alignment algorithm and with an E-value of 1E-5, using three models from the Pfam database (http://pfam.xfam.org/, accessed on 23 November 2020) [40], including two RING finger domains (PF13445 and PF14634) and one B-box domain (PF00643) as templates, respectively. The intersection of the three produced hmm search result files were then extracted with a shell script and submitted to the Simple Modular Architecture Research Tool (SMART, http://smart.embl-heidelberg.de, accessed on 27 November 2020) [41] for domain analysis. According to the domain architecture results, redundant TRIMs were manually filtered out. Finally, grass carp TRIMs (*Ci*TRIMs) were identified according to the criterion of whether they possessed conserved RBCC or RB domain assemblies.

### 2.2. Gene Structure Analysis and Subcellular Localization Prediction of CiTRIMs

The information of the amino acid sequence length and number of introns and exons for *Ci*TRIMs was extracted from the grass carp genome annotation file by using a shell script. The protein molecular weights and isoelectric points of the *Ci*TRIMs were predicted through Expasy (https://www.expasy.org/, accessed on 10 December 2020) [42]. The amino acid sequences of the *Ci*TRIMs were submitted to the online platform Euk-mPLoc 2.0 Server (http://www.csbio.sjtu.edu.cn/bioinf/Cell-PLoc-2/, accessed on 11 December 2020) [43] for subcellular localization prediction.

### 2.3. Domain/Motif Architecture and the Dendrogram of CiTRIMs

The domain architecture results for the *Ci*TRIMs were collected from SMART and then visualized using Adobe Illustrator 2020 (version 24.1.0). The protein sequences of the *Ci*TRIMs were submitted to the MEME Suite 5.3.3 (http://meme-suite.org/tools/meme, accessed on 21 December 2020) [44] with the application of Motif Discovery for motif analysis and with the parameter of 10 for selecting the number of motifs. The dendrogram of the *Ci*TRIMs was constructed as previously described [45]. Multiple amino acid sequence alignments of the *Ci*TRIMs were conducted using the ClustalW 1.81 software with the default parameters. The MEGA 6.06 software [45] was then used to construct a dendrogram with the neighbor-joining algorithm and with the parameters including the p-distance, complete deletion and gap setting; the results were tested for reliability over 1000 bootstrap replicates, after which the editing was carried out online by using EVOLVIEW (https://evolgenius.info, accessed on 23 December 2020) [46].

### 2.4. Chromosomal Localization and Collinearity Analysis

The chromosomal localization analysis of *Ci*TRIMs was conducted according to the previous method with slight modifications [47]. In brief, the chromosome map draft was redrawn by mapping the assembled 301 scaffolds (with an average length of >179,941 bp) from the published grass carp genome into chromosomes. The number and localization information for the *Ci*TRIMs on the chromosomes was obtained using a shell script and then visualized using Mapgene2Chromosome (version 2.1) [48]. The gene collinearity analysis of the *Ci*TRIMs was also performed according to the previous methods. The human genome (GRCh38) and zebrafish genome (GRCz11) data were downloaded from the Ensembl Animal Genome database (http://www.ensembl.org/index.html, accessed on 3 January 2021) [49]. The TBtools software was used to handle the redundancies in the grass carp, zebrafish, and human genomes [50]. The longest transcript sequence for each gene in these three de-redundant genomes was extracted as the representative sequence using TBtools. The gene collinearities of TRIMs from grass carps, zebrafish and humans were analyzed by using multiple Collinear Scanning Toolkits (MCScanX) [51].

### 2.5. Expression Analysis of CiTRIMs in Uninfected Grass Carp Tissues

To investigate the tissue expression patterns of the *Ci*TRIMs, the published transcriptome data for uninfected grass carp tissues, including the kidneys, liver, head kidneys, spleen, brain, and embryo, were downloaded from GCGD. The protein sequences of the *Ci*TRIMs from the grass carp genome were submitted to the online platform eggNOG-MAPPER (http://eggnog-mapper.embl.de/, accessed on 4 January 2021) [52] with default parameters for genome-wide functional annotation. The RPKM (reads per kilobase per million mapped reads) values for the *Ci*TRIMs were obtained from the transcriptome data and then submitted to TBtools for normalization and the production of a heatmap.

### 2.6. Expression Analysis of CiTRIMs in Spleen Tissue during GCRV Infection 

The published transcriptome raw data (SRP095827) [53] for the spleens from grass carp infected with GCRV on days 1, 3, 5 and 7 were downloaded from the Sequence Read Archive (SRA, https://www.ncbi.nlm.nih.gov/sra/?term=, accessed on 17 January 2021) database for identifying *Ci*TRIMs differentially expressed during GCRV infection. These raw data were reanalyzed with the following workflow: TrimGalore (version 0.6.4) was first used to eliminate adapter and low-quality sequences from the raw reads with the parameters of -q 20, -phred 33, -stringency 2, -length 20 and -e 0.1. FastQC (version 0.11.8) was also adopted to assess whether the cleaned reads met the requirements for subsequent analyses. Then, Hisat2 (version 2.1.0) [54] was employed to align cleaned reads to the grass carp genome with default parameters, followed by the counting of transcripts using FeatureCount (version 1.6.4) [55]. A differential expression analysis was performed using the DESeq R package (version 1.30.1) [56] with default parameters. The Benjamini and Hochberg approach was used to control the false discovery rate (FDR) through the adjustment of the resulting *p*-values. The average RPKM values for the *Ci*TRIMs from three biological replicates were obtained from the transcriptome data and submitted to TBtools for normalization and the production of a heatmap. The differentially expressed *Ci*TRIMs were identified in terms of fold changes > 2 and FDRs (or adjusted *p-*values) < 0.05.

WGCNA (version 1.70-3) was used to further explore if the differentially expressed *Ci*TRIMs were associated with immune processes based on the transcriptomes of spleens from grass carp infected with GCRV on Days 1, 3, 5 and 7, using the previous method with slightly modifications [57]. In brief, the function of genefilter’s varFilter (version 1.72.1) [58] in the R package was used to exclude the genes with low expression variation within samples, with a var.cutoff of 0.3. The soft-thresholding power was selected by using the function pickSoftThreshold; then, the function blockwiseModules was adopted for gene network construction and module identification, with the parameters of corType = pearson, power = 6, networkType = unsigned, TOMType = unsigned, maxBlockSize = 100,000 and other default parameters, followed by the calculation of the coefficients of the correlation between module and trait (infection time points) by using the function cor. The Student asymptotic *p*-value was determined using the function of corPvalueStudent, with an Student asymptotic *p*-value < 0.05, marking a significant difference. The targeted module was exported from the Cytoscape software using the function of exportNetworkToCytoscape and with a threshold of 0.415. Finally, highly interconnected gene networks including differentially expressed *Ci*TRIMs were obtained by the application of MCODE within Cytoscape, followed by a GO enrichment analysis of functional annotations including Biological Process, Cellular Component and Molecular Function with Metascape (https://metascape.org/gp/index.html#/main, accessed on 3 February 2021) [59].

### 2.7. Expression Analysis for CiTRIMs in Kidney Cell Line during GCRV Infection

The published transcriptome raw data (PRJNA597582 and PRJNA597542) [60] of the grass carp kidney cell line (CIK) after GCRV challenge at 0 h (control), 6 h, 12 h and 24 h were also downloaded for identifying *Ci*TRIMs differentially expressed during GCRV infection. These raw data were reanalyzed with the same workflow as described above. The average RPKM values for the *Ci*TRIMs from three biological repeats at each infection time point were obtained from the transcriptome data and then submitted to TBtools for normalization and the production of a heatmap. The differentially expressed *Ci*TRIMs were identified in terms of fold changes > 2 and adjusted *p*-values (or FDRs) < 0.05.

To further explore if the differentially expressed *Ci*TRIMs were associated with immune processes in the CIK transcriptomes, a gene expression trend analysis was conducted using Short Time-series Expression Miner (STEM, version 1.3.13), with reference to the method previously described [61]. Briefly, the medians of the differentially expressed genes’ RPKM values from the CIK transcriptomes were first taken and imported into STEM to analyze the gene expression trends with the parameter of log normalize data. Different profiles where specific gene clusters showed similar expression trends were produced. The significantly similar gene expression trends were indicated as *p*-values < 0.05 by STEM. Targeted profiles containing differentially expressed *Ci*TRIMs were exported and submitted to Metascape, followed by a GO enrichment analysis with the zebrafish annotation database as the reference [59].

### 2.8. The Verification of Differentially Expressed CiTRIMs by qPCR

CIK cells were cultured in an incubator (Thermo Fisher Scientific, Waltham, MA, USA) at 28 °C with 5% CO_2_ and with Medium 199 (Gibco, Grand Island, NY, USA) liquid medium containing a 1% penicillin–streptomycin mixture and 10% fetal bovine serum. When covering 80% of the bottom of the culture flask (Corning, NY, USA), the cells were detached using trypsin and transferred into 6-well plates (Corning, NY, USA). For the GCRV challenge experiment, GCRV (GCRV JX-01 strain, kindly provided by Professor Zeng Lingbing from the Yangtze River Fisheries Research Institute of the Chinese Academy of Fishery Sciences) suspension was added into the 6-well plates. The cell samples were collected after the GCRV challenge at 0 h, 6 h, 12 h and 24 h. Three biological replicate samples were taken for each infection time point.

Total RNA from the cell samples was extracted using an RNA-easy^TM^ Isolation Reagent Kit (Vazyme, Nanjing, China), according to the manufacturer’s instructions, followed by cDNA synthesis with a RevertAid First Strand cDNA Synthesis Kit (Thermo Fisher Scientific, Waltham, MA, USA). Specific primers (Table 1) were designed to detect the mRNA expression levels of genes, including eleven differentially expressed *Ci*TRIMs and interferon regulatory factor 3 (*Ci*IRF3) identified in transcriptomes, and VP2, which represented a GCRV protein component, by quantitative real-time polymerase chain reaction (qPCR) using a CFX96 Touch Real-Time PCR Detection System (Bio-Rad, Hercules, CA, USA). The grass carp β-actin gene was employed as the internal control. The amplifications were performed in triplicate in a total volume of 10 µL, containing 5 µL of ChamQ^TM^ Universal SYBR qPCR Master Mix (Vazyme, Nanjing, China), 1 µL of diluted cDNA, 0.4 µL of each primer and 3.2 µL of ddH_2_O. The cycle conditions were as follows: 1 cycle at 95 °C for 3 min, 40 cycles at 95 °C for 15 s, 60 °C for 15 s and 72 °C for 15 s. The relative expression levels of the genes were analyzed with the Ct method (2^−ΔΔCt^ method) [62]. The data are expressed as means ± standard deviations and were analyzed with the Statistical Package for Social Sciences Version 25.0 (SPSS Inc., Chicago, IL, USA). The significance of the differences in expression levels was tested by one-way analysis of variance (ANOVA) and multiple comparisons. Statistically significant differences were represented by *p* < 0.05.

## 3. Results

### 3.1. Genome-Wide Identification of CiTRIMs

TRIMs are characterized by RBCC or RB domain assemblies [9]. A total of 42 *Ci*TRIMs were identified in the grass carp genome with hmmsearch and SMART analysis according to this criterion (Table 2). Among them, 37 *Ci*TRIMs were named and numbered with reference to their homologous counterparts from the genomes of zebrafish and humans based on sequence similarity and identity, while five *Ci*TRIMs, including *Ci*TRIM35-50, *Ci*TRIM39-like, *CiTRIM*111, *Ci*TRIM112 and *Ci*btr40, whose homologs were not identified in the genomes from zebrafish and humans, and could be found in the teleost genomes of Pimephales promelas and Sinocyclocheilus anshuiensis by BLAST search (Table 2), were given names referring to the nomenclature previously described [10].

Forty-two *Ci*TRIMs were structured with different numbers of introns and exons (Table 2). Their coding sequence and encoded amino acid sequence lengths were 813~3978 bp and 271~1326 aa, respectively (Table 2). The proteins of the *Ci*TRIMs were predicted with molecular weights (MWs) ranging from 14.64 to 89.11 KDa (Table 2). A total of 26 *Ci*TRIMs were acidic, with isoelectric points (PIs) ranging from 4.87 to 6.65, and 16 *Ci*TRIMs were alkaline, with PIs ranging from 7.53 to 8.73 (Table 2). Subcellular localization prediction showed that *Ci*TRIMs tended to be located in the cytoplasm, cytoskeleton and nucleus, with 35 *Ci*TRIMs in the cytoplasm and 17 *Ci*TRIMs in other multiple regions of the cell, among which only two *Ci*TRIMs (*Ci*TRIM18 and *Ci*TRIM55b) were located in the cytoskeleton (Table 2).

### 3.2. Dendrogram and Structural Features of CiTRIMs

**Figure 1:**
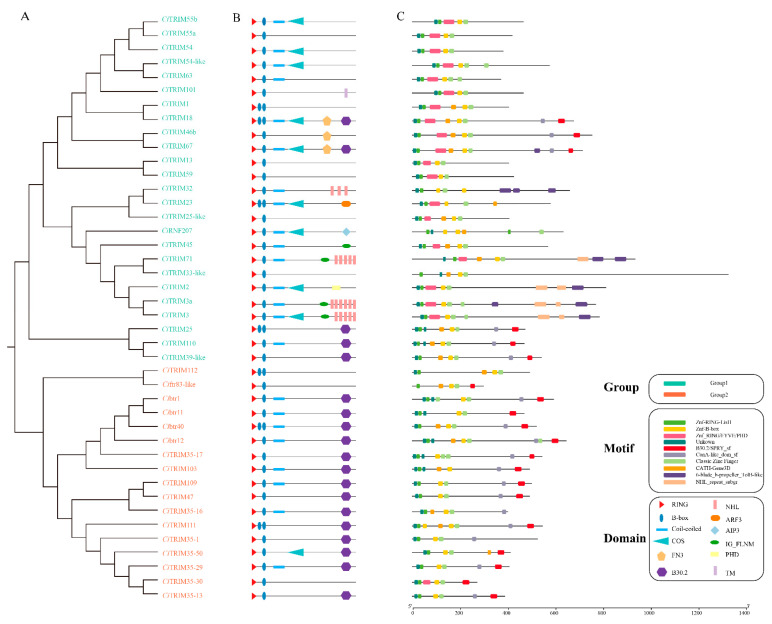
Dendrogram, domains and motifs of *Ci*TRIMs. (**A**) The dendrogram of *Ci*TRIMs was divided into two large branches, indicated in green and red. (**B**) The schematic diagram of domain architectures of *Ci*TRIMs. (**C**) Conserved motifs of *Ci*TRIMs predicted by MEME. The scale on the bottom margin was measured by amino acid number.

According to the topological structure of the dendrogram, 42 *Ci*TRIMs could be divided into two major branches, with 25 *Ci*TRIMs in Group 1 and 17 *Ci*TRIMs in Group 2 (Figure 1A). A total of 24 *Ci*TRIMs harbored conserved RBCC domain assemblies and the other *Ci*TRIMs harbored conserved RB domain assemblies at their N-terminal; on the other hand, the C-terminal domains of the *Ci*TRIMs, especially those in Group 1, were quite varied (Figure 1B); they consisted of eight categories of domains, including the COS (C-terminal subgroup one signature) domain, TM (transmembrane) domain, FN3 (fibronectin type 3) domain, B30.2 domain, ARF3 (ADP-ribosylation factor 3) domain, AIP3 (actin interacting protein 3) domain, filamin (filamin-type immunoglobulin) domain and PHD (plant homeodomain) domain. Most of the *Ci*TRIMs from Group 2 harbored only the B30.2 domain at the C-terminus, except for *Ci*TRIM112, *Ci*ftr83-like and *Ci*TRIM35-30 (Figure 1B). In total, 19 TRIMs-B30.2 were identified in the grass carp genome (Figure 1B). Meanwhile, 10 conserved motifs in *Ci*TRIMs were predicted by MEME Suite 5.3.3. The results showed that all the *Ci*TRIMs had the two motifs Znf-RING_LisH and Znf-B-box at their N-terminal motif architectures (Figure 1C), while the C-terminal motif architectures of the *Ci*TRIMs, especially those in Group 1, were significantly diversified (Figure 1C).

### 3.3. Chromosomal Location of CiTRIMs

**Figure 2:**
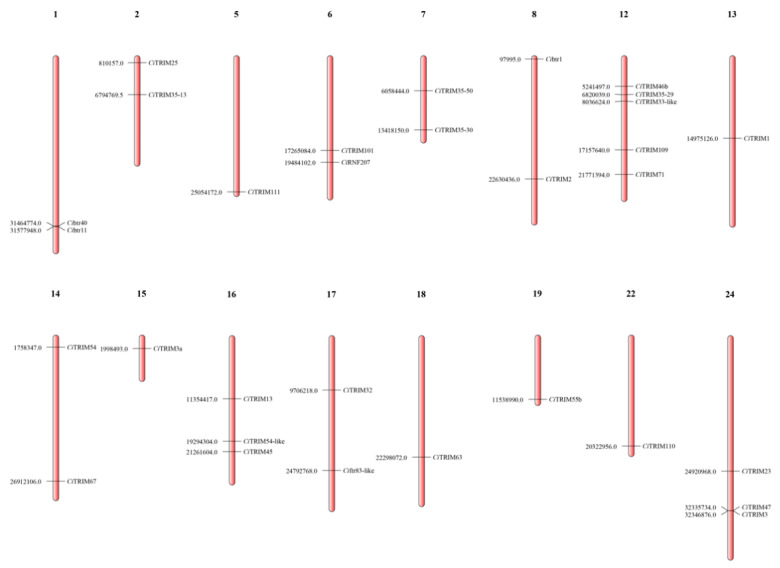
Chromosomal locations of *Ci*TRIMs. The number on the left represents the position of the *Ci*TRIM gene on the chromosome.

A total of 301 scaffolds (with an average length of >179,941 bp) from the grass carp genome were assembled into 24 chromosomes by Mapgene2Chromosome (V2.1), with 114 scaffolds anchored on linkage groups (Figure 2). Only 31 out of 42 *Ci*TRIMs were discovered on 16 chromosomes. The other 11 *Ci*TRIMs might have been lost due to low-quality chromosome assembly. No *Ci*TRIM was found on chromosomes 3, 4, 9, 10, 11, 20, 21 and 23, while five *Ci*TRIMs were located on chromosome 12, which possessed the largest number of *Ci*TRIMs (Figure 2). It has been reported that teleost TRIMs undergo massive expansion mainly though tandem repeats to adapt to environmental changes during evolution [22]. The possible presence of tandem repeats in the *Ci*TRIMs was investigated with MCScanX, but none were found (data not shown).

### 3.4. Gene Collinearities of CiTRIMs with TRIMs from Zebrafish and Humans

**Figure 3:**
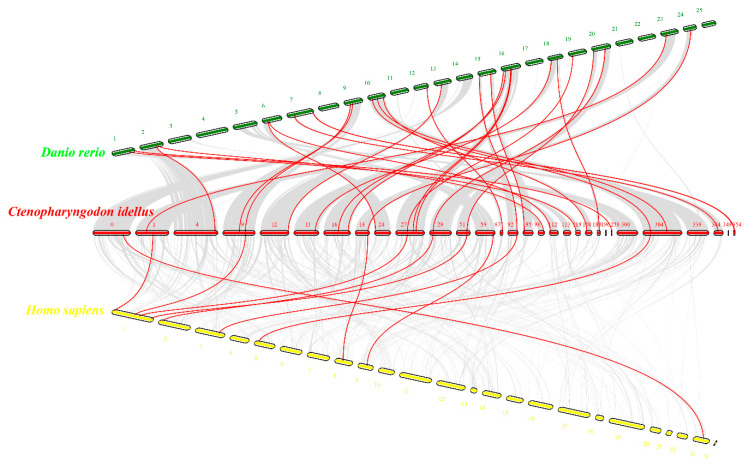
Gene collinearity analysis of *Ci*TRIMs with those from the zebrafish and human genomes. Red, green, and yellow short sticks represent scaffolds from the grass carp genome, zebrafish genome and human genome, respectively. The red lines indicate TRIM gene pairs between species.

To further understand the evolutionary features of *Ci*TRIMs, the gene collinearities of *Ci*TRIMs with TRIMs from zebrafish and humans were analyzed using MCScanX. A total of 42 *Ci*TRIMs were distributed on 30 different scaffolds of the grass carp genome (Figure 3). According to the results of the collinearity analysis, 30 pairs of homologous TRIMs were identified between the grass carp genome and zebrafish genome, while 10 pairs of homologous TRIMs were identified between the grass carp genome and human genome (Figure 3). No homologous TRIMs were detected in the zebrafish and human genomes for five *Ci*TRIMs, including *Ci*TRIM35-50, *Ci*TRIM39-like, *Ci*TRIM111, *Ci*TRIM112 and *Ci*btr40 (Figure 3).

### 3.5. Tissue Expression Patterns of CiTRIMs

**Figure 4:**
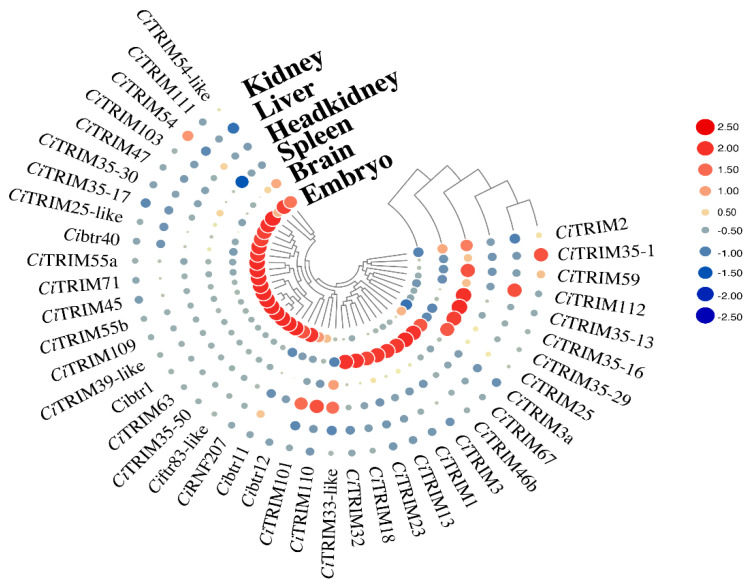
Heatmap based on expression profiles of *Ci*TRIMs in six tissues from uninfected grass carp including kidneys, liver, head kidneys, spleen, brain, and embryo. The sizes of red or blue dots in the heatmap reflect gene expression levels, and the color shades represent the cluster correlations of genes in terms of their expression levels.

To clarify the tissue expression patterns of *Ci*TRIMs, the transcriptomes of the kidneys, liver, head kidneys, spleen, brain, and embryo from uninfected grass carp were analyzed. As presented in Figure 4, 21 *Ci*TRIMs, including *Ci*TRIM103, *Ci*btr40 and *Ci*TRIM55a, showed high mRNA expression levels in the embryos, and nine *Ci*TRIMs, including *Ci*TRIM13, *Ci*TRIM18 and *Ci*TRIM23, were highly expressed in the brain. Six *Ci*TRIMs (including *Ci*TRIM13, *Ci*TRIM18 and *Ci*TRIM23), three *Ci*TRIMs (*Ci*TRIM101, *Ci*TRIM110 and *Ci*TRIM33-like), two *Ci*TRIMs (*Ci*TRIM35-1 and *Ci*TRIM54) and *Ci*TRIM112 were mainly expressed in the spleen, head kidneys, kidneys, and liver, respectively, which indicated the specific and high expression of certain *Ci*TRIMs that occurred in the immune tissues of uninfected grass carp.

### 3.6. Expression Patterns of Potential Antiviral CiTRIMs in Grass Carp Spleen Tissue during GCRV Infection

**Figure 5:**
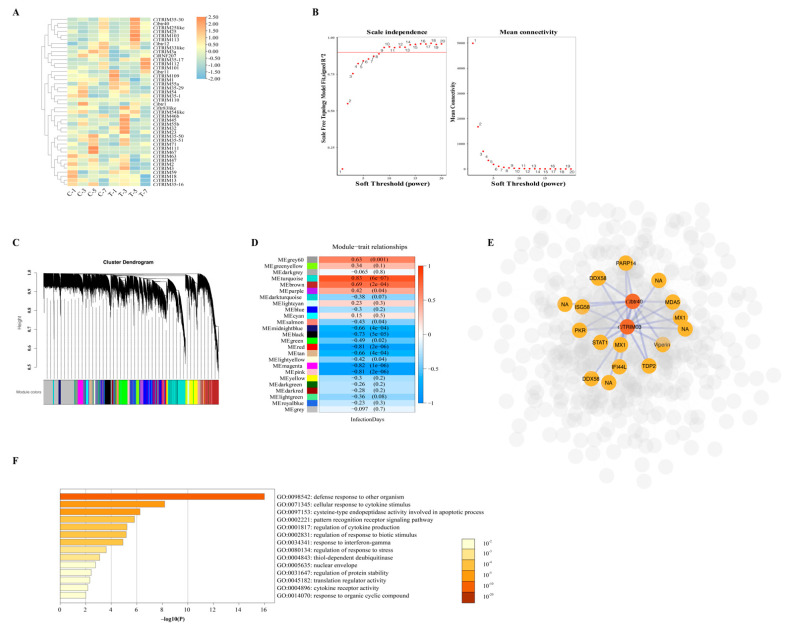
Transcriptome analyses of the spleens from grass carp infected with GCRV within 7 days. (**A**) Heatmap of 42 *Ci*TRIMs’ expression changes in spleen after GCRV infection on days 1, 3, 5 and 7. C-1: control group, day 1; C-3: control group, day 3; C-5: control group, day 5; C-7:control group, day 7; T-1: treatment group, day 1; T-3: treatment group, day 3; T-5: treatment group, day 5; T-7: treatment group, day 7. (**B**) Analysis of network topology for various soft-thresholding powers. The left panel shows the scale-free fit index (y-axis) as a function of the soft-thresholding power (x-axis). The right panel displays the mean connectivity (degree, y-axis) as a function of the soft-thresholding power (x-axis). (**C**) Clustering dendrogram of genes, with dissimilarity based on topological overlap, together with assigned module colors, that contain a cluster of genes with similar biological functions. (**D**) Pearson correlation analysis of module and trait (infection time points). Each cell contains the Pearson correlation coefficient and Student asymptotic *p*-value. The correlation intensity color is illustrated by the legend on the right. (**E**) Gene co-expression network in brown module. *Ci*btr40, *Ci*TRIM103 and 12 genes, including DDX58 (RIG-I-like receptor), PKR, Viperin, MDA5, STAT1, MX1 and ISG58, with the highest node degree distribution value were highlighted in the network. (**F**) GO enrichment analysis of genes in the brown module using Metascape. Only top 14 enriched GO terms are shown. The color shade reflects the significance (*p*-value) of the GO enrichment analysis, which is represented as –log10(P) on the bottom coordinate ruler (x-axis).

To identify *Ci*TRIMs differentially expressed during GCRV infection, the transcriptomes of the spleens in grass carp after GCRV challenge on Days 1, 3, 5 and 7 were analyzed. Although various *Ci*TRIMs were slightly upregulated after GCRV challenge at each time point, most of their expression levels showed no significant difference (*p* > 0.05), compared to those in the control (Figure 5A). Only three *Ci*TRIMs, namely, *Ci*btr40, *Ci*TRIM103 and *Ci*TRIM112, were significantly differentially expressed in the transcriptome of the spleen in grass carp after GCRV challenge on the fifth day, compared to the expression in the control (*p* < 0.05; Figure 5A).

WGCNA was used to explore the related biological functions of three significantly differentially expressed *Ci*TRIMs based on the spleen transcriptomes during GCRV infection. A soft-threshold power that was the most suitable for the construction of a gene co-expression network was chosen from a list of 1–20 candidate powers (Figure 5B). Then, the dynamic hybrid cleavage method was adopted to merge gene clusters with co-expression into the specific modules on the same branch (Figure 5C). After removing the genes with low gene expression variation, a total of 22,924 genes were obtained and clustered into 26 modules (Figure 5D). Except for the gray module, the turquoise module possessed the largest number of genes, with a total of 2883 co-expressed genes, while the dark-gray module harbored the fewest genes, with a total of 31 co-expressed genes (data not shown). After the Pearson correlation analysis of 26 modules with trait (the infection time points), it was found that the gene cluster in the turquoise module showed the highest positive correlation coefficient (0.83), followed by that in the brown module (0.69), while the gene cluster in the magenta module represented the highest negative correlation coefficient (−0.82; Figure 5D). Since all the three differentially expressed *Ci*TRIMs were clustered in the brown module, this module was selected for subsequent analyses. When filtering the undirected network with an adjacency threshold > 0.45 in the brown module using Cytoscape, *Ci*TRIM112 was excluded. The filtered network showed that both *Ci*TRIM103 and *Ci*btr40 were linked with the hub gene DEAD (Asp-Glu-Ala-Asp) Box Polypeptide 58 (DDX58) (alternatively named retinoic acid-inducible gene I (RIG-I)-like receptor), as well as several vital type I IFN response pathway genes, such as melanoma differentiation associated gene 5 (MDA5), signal transducer and activator of transcription 1 (STAT1), IFN stimulating gene 58 (ISG58), double-stranded RNA-dependent protein kinase (PKR), myxovirus 1 (MX1) and viperin (Figure 5E). The GO enrichment analysis of this gene network revealed that the top three enriched GO terms were associated with immune defense processes, including defensive responses to other organisms, cellular responses to cytokine stimulation and cysteine-type endopeptidase activity involved in apoptosis (Figure 5F).

### 3.7. Expression Patterns of Potential Antiviral CiTRIMs in CIK during GCRV Infection

**Figure 6:**
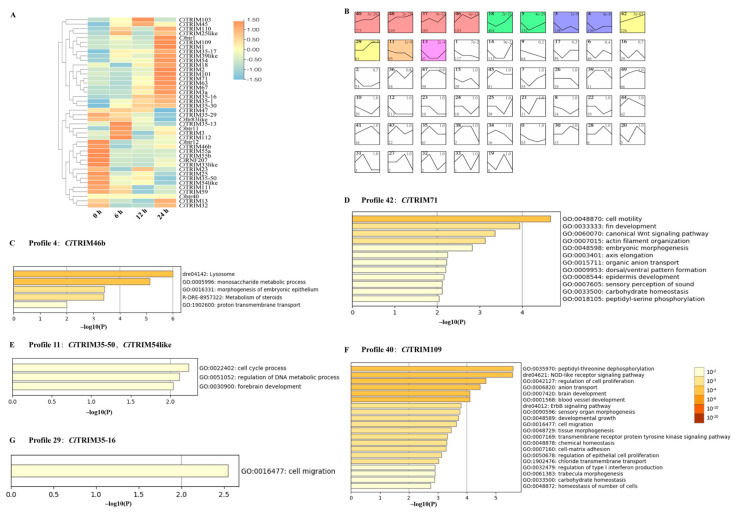
Transcriptome analyses of CIK after GCRV challenge. (**A**) Heatmap of 42 *Ci*TRIMs’ expression changes in CIK after GCRV infection at 0 h, 6 h, 12 h and 24 h. (**B**) Expression trend for genes in CIK transcriptomes during GCRV infection. A total of 50 profiles where the gene cluster exhibited a similar expression trend after GCRV challenge at 0 h, 6 h, 12 h and 24 h were produced using STEM. The data in the top-left corner, top-right corner and top-right corner represent the profile ID, the significance of cluster correlation and the number of genes in profiles, respectively. GO enrichment analysis for genes including differentially expressed *Ci*TRIMs in Profile 4 (**C**), Profile 42 (**D**), Profile 11 (**E**), Profile 40 (**F**) and Profile 29 (**G**). Coordinate ruler on the bottom (x-axis) of profiles represents the significance (*p*-value) from GO enrichment analysis, which is shown as –log10(P).

The transcriptomes of CIK after GCRV challenge at 6 h, 12 h and 24 h were also analyzed to survey the *Ci*TRIMs differentially expressed during GCRV infection. A total of 4167 differentially expressed genes were identified. Among the 42 identified *Ci*TRIMs, eight whose expression levels showed significant differences at 6 h, 12 h or 24 h compared to the control (0 h) were determined as the differentially expressed ones (*p* < 0.05; Figure 6A). Among these differentially expressed *Ci*TRIMs, *Ci*TRIM46b was significantly down-regulated at 6 h; *Ci*TRIM35-16 was significantly upregulated at 12 h and 24 h; *Ci*TRIM109 was significantly upregulated at 6 h and 24 h; *Ci*TRIM2, *Ci*TRIM71 and *Ci*TRIM110 were significantly upregulated at 24 h; and *Ci*TRIM35-50 and *Ci*TRIM54-like were significantly down-regulated at 24 h after GCRV challenge, compared to control (*p* < 0.05; Figure 6A). 

Gene expression trend analysis using STEM was performed to further reveal the biological functions of these eight differentially expressed *Ci*TRIMs based on the CIK transcriptomes during GCRV infection. All the differentially expressed genes from the CIK transcriptomes were classified into fifty clusters and formed fifty profiles (numbered from 1 to 50), where the gene cluster exhibited a similar expression trend after GCRV challenge at 0 h, 6 h, 12 h and 24 h (Figure 6B). The expression trends of gene clusters in 12 profiles with colored backgrounds were significantly similar when analyzed by STEM (*p*-value < 0.05; Figure 6B). Profile 40 included the largest number of genes, with a total of 775 genes (Figure 6B). Six out of eight differentially expressed *Ci*TRIMs were, respectively clustered in five profiles where the gene expression trend was significantly similar (*p*-value < 0.05; Figure 6B). In detail, *Ci*TRIM35-50 and *Ci*TRIM54-like were clustered in Profile 11, *Ci*TRIM109 was clustered in Profile 40, *Ci*TRIM46b was clustered in Profile 4, *Ci*TRIM35-16 was clustered in Profile 29 and *Ci*TRIM71 was clustered in Profile 42 (Figure 6B). All the genes within these five profiles were imported into Metascape for GO enrichment analysis. The results reveal that the gene clusters in these five profiles were annotated with different GO terms (Figure 6C–F). Notably, the gene cluster including *Ci*TRIM109 in Profile 40 was enriched with a total of 20 GO terms, two of which were tightly associated with antiviral immune defense processes, including the NOD-like receptor signaling pathway and regulation of type I interferon production (Figure 6F).

### 3.8. The Verification of Differentially Expressed CiTRIMs during GCRV Infection

**Figure 7:**
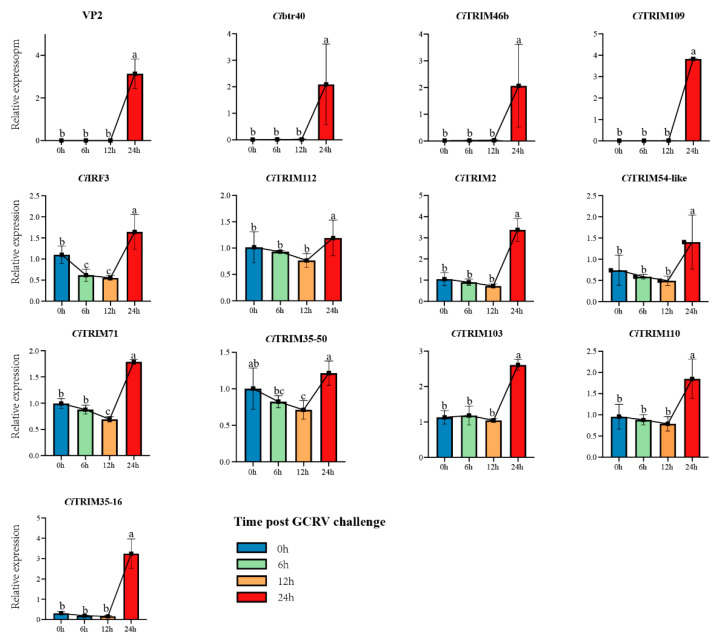
Expression profiles of VP2, *Ci*IRF3 and 11 *Ci*TRIMs in response to GCRV challenge in CIK. Expression data were normalized using β-actin as internal control; error bars indicate standard deviations among three biological replicates. The letters (a, b and c) indicate significant differences among expression levels at different time points after GCRV challenge (*p* < 0.05).

Eleven differentially expressed *Ci*TRIMs as well as several type I IFN response pathway genes were identified in the two sets of transcriptomes above. To verify their gene expression, an experiment in which CIK cells were challenged with GCRV over 24 h was conducted. The sequential expression changes of *Ci*IRF3 (an important IFN regulatory factor) [63], VP2 (a protein component of GCRV) [64] and these 11 differentially expressed *Ci*TRIMs were detected by qPCR to explore the correlation of the gene expression trends between *Ci*IRF3/VP2 and differentially expressed *Ci*TRIMs and to reveal the process of host–pathogen interaction during GCRV infection. The results show that the mRNA expression level of VP2, which indicated GCRV replication [64], was significantly upregulated after challenge with GCRV at 24 h, compared to that at 0 h (*p* < 0.05; Figure 7). Meanwhile, *Ci*IRF3, along with eleven differentially expressed *Ci*TRIMs identified in the two sets of transcriptomes above, was also significantly upregulated after GCRV challenge at 24 h (*p* < 0.05; Figure 7). In addition, three *Ci*TRIMs, including *Ci*btr40, *Ci*TRIM46b and *Ci*TRIM109, showed similar expression trends to VP2, all of which were little expressed after GCRV challenge at 0 h, 6 h and 12 h and sharply upregulated at 24 h (*p* < 0.05; Figure 7). The expression trends for eight other *Ci*TRIMs were similar to the trend for *Ci*IRF3 after GCRV challenge at 0 h, 6 h, 12 h and 24 h, all of which first decreased and then increased, with peak expression at 24 h (Figure 7). Moreover, the expression levels of *Ci*btr40, *Ci*TRIM103 and *Ci*TRIM109, which all belonged to TRIMs-B30.2 and were linked with the type I IFN response pathway by WGCNA and the gene expression trend analysis based on the transcriptome data, were significantly upregulated by 235-fold, 2-fold, and 916-fold after GCRV challenge at 24 h, respectively, compared to at 0 h (*p* < 0.05; Figure 7).

## 4. Discussion

The TRIM protein family is a class of proteins that possess conserved RBCC or RB domain assemblies at their N-termini and a variety of domains at their C-termini, widely existing in the majority of metazoa, which play multiple roles in physiological or pathological processes such as growth and development [65], metabolism and autophagy [12], transcriptional regulation [13], carcinogenesis [14] and antiviral immunity [15]. During the long-term evolution of animals, large differences in TRIM family gene numbers have arisen among species. Compared to invertebrates, vertebrates seem to possess, overall, a larger number of TRIMs. For example, humans have a total of 69 TRIMs, which is more than three times the number of TRIMs in nematodes, *Caenorhabditis elegans* [4]. In teleosts, two or more rounds of whole genome duplication can be observed [66,67], resulting in more universal variation in the numbers of TRIMs among species. The Ballan wrasse (*Labrus bergylta*) from Perciformes seems to have the largest number, with 369 extant TRIMs; the Red-bellied piranha (*Pygocentrus nattereri*) from Characiformes possesses 229 TRIMs, and the tiger tail seahorse (*Hippocampus comes*) from Gasterosteiformes only harbors 62 TRIMs [68]. The number of TRIMs may reflect the evolutionary processes or divergence times of species, making the identification of TRIMs an attractive topic for teleosts.

Recently, different software and methods have been employed to identify TRIMs in teleosts. Sardiello et al. combined the software packages PHI-BLAS and TBLASTN to identify a total of 240 TRIMs in zebrafish based on NR databases [4]. Zhang et al. used the blast method to identify a total of 196 zebrafish TRIMs in the NCBI and Ensembl databases, with the sequence encoding the RBCC domain assemblies as the bait [69], while Boudinot et al. refined the number of zebrafish TRIMs to 208 in the Ensembl databases by using the Hidden Markov Model with RING finger and B-box domains as the templates and with the domain architectures of human TRIMs as the reference to exclude redundancy [10]. These studies indicate that using different methods and databases may identify different numbers of TRIMs even among the same species. In the present study, we adopted the Hidden Markov Model to identify 42 *Ci*TRIMs in the grass carp genome with a conserved B-box domain and two conserved RING finger domains as the templates and according to the criterion of whether they possessed RBCC or RB domain assemblies. The authenticity of the identified *Ci*TRIMs was further verified by gene collinearity and chromosomal location analyses. On the other hand, we also tried to use our method in other species to test its specificity and sensitivity. In humans, for example, we found 267 TRIMs containing the RBCC or RB domain assemblies from 354 TRIM transcripts in genome annotation files (GRCh38.p13), which represented results consistent with those of a previous study [9]. A total of 106 TRIM genes were annotated in the grass carp genome [39]. Our results implied that 64 TRIM genes annotated in the grass carp genome did not harbor the N-terminal conserved RBCC or RB domain assemblies. To our knowledge, both the annotations for 354 TRIM transcripts in humans and 106 TRIM genes in grass carp tend to be obtained through sequence alignments against multiple databases, which would cause false positive annotations [9,39]. Although several *Ci*TRIMs identified in the previous study were not found in our study, two reasons may explain this difference based on reviewing multiple studies on the identification of zebrafish TRIMs. One reason is that different template sequences and search methods were used in the present and previous studies. Luo et al. utilized a set of zebrafish ftr gene sequences as templates to search all the putative ftrs in grass carp by using the blast method, while our study sought to identify all the *Ci*TRIMs based on conserved domains across species using the Hidden Markov Model [70]. Using the domain architectures of zebrafish and human TRIMs as the reference to exclude redundancy, we identified at least seven putative *Ci*ftrs, fewer than the previous study identified [70]. Additionally, the databases used in the identification of TRIMs also differ between the present and previous studies [70]. Luo et al. chose the NCBI and Ensembl databases, as well as the non-referenced transcriptomes, for retrieving the TRIMs in grass carp [70], while, in this study, we only downloaded the grass carp genome as the search database for the identification of *Ci*TRIMs, which could effectively reduce false positive results. Nevertheless, we realize that several *Ci*TRIMs have still not been identified through our subsequent gene tandem repeat analysis, probably due to the assembly of the extant genome (only with the contigs N50 of 40,781 bp). It is believed that a higher quality genome is required for identifying all the *Ci*TRIMs containing intact RBCC or RB domain assemblies, since Boudinot et al. found more zebrafish TRIMs in the genome Version Z9 than that in the genome Version Z8 [10]. 

The C-terminal domains help to build interactions with other proteins and often determine the functional specificity in TRIMs [16]. To date, more than 11 C-terminal domains have been identified in TRIMs, which perform a variety of biological functions [16,71]. For example, a deficiency of the C-terminal domains of COS, FN3 and B30.2 in TRIM18 weakens the activation of the mechanistic target of rapamycin complex 1 (mTORC1) signaling, causing abdominal midline dysplasia in the fetus [72]. The C-terminal domains of PHD and BROMO in TRIM24 can bind to chromatin and serve as potential therapeutic targets for breast cancer [73]. The filamin and NHL domains of TRIMs play important roles in neuronal differentiation [11]. The ARF domain at the C-terminus in TRIM23 appears to trigger autophagy by activating TBK1 through the non-traditional ubiquitinated GTPase [74]. In vertebrates, the B30.2 domain is the most frequent at the C-termini of TRIMs [4]. In humans, there are a total of 35 TRIM-B30.2s [16], 30 of which have been proved to participate in immune responses [16,75]. Distinct tissue expression patterns also reflect the functional differentiation of TRIMs. For instance, when TRIM4 is highly expressed in nervous tissues, neural tube defects are detected [76], while the overexpression or knockdown of TRIM4 in immune tissues significantly affects the expression of IRF3, nuclear factor-kappa B (NF-κB) and IFN, demonstrating its involvement in antiviral immunity in humans [77]. Such evidence indicates that different tissue expression patterns and C-terminal domains confer TRIMs with functional diversity. In the present study, the expression analysis showed that 42 *Ci*TRIMs were obviously expressed in different tissues. Specifically, nine, six, three, two and one *Ci*TRIMs were mainly expressed in the brain, spleen, head kidneys, kidneys, and liver, respectively. The structural analyses also identified eight C-terminal domains, including the COS domain, B30.2 domain, FN3 domain, TM domain, NHL domain, ARF3 domain, AIP3 domain, filamin domain and PHD domain in *Ci*TRIMs, implying the functional differentiation of grass carp TRIMs. In addition, 19 TRIMs-B30.2 were found, with the largest number in *Ci*TRIMs (accounting for ~45.2%), and they were clustered into a distinct branch in the dendrogram, suggesting that grass carps have also evolved with a cluster of TRIMs linked to immune defense, similar to other vertebrates. 

Although the expansion of the B30.2 domain is common in vertebrates, TRIM-B30.2 family members differ among various species [4]. Thus, almost every species has specific TRIM-B30.2 family members; for example, 44% of TRIMs contain the B30.2 domain in humans; 55%, in puffer fish; and 83%, in zebrafish [10,16]. In teleosts, three major subfamilies of TRIMs-B30.2 have developed, including hltrs, btrs and ftrs, via the massive duplication of three ancestor TRIM-B30.2 genes, while no corresponding duplication phenomena are found in other vertebrate species [10]. In fact, teleost hltrs and btrs are produced by the duplication of TRIM35 and TRIM39, respectively, both of which play important roles in immune responses [10,21]. TRIM35 can directly mediate polyubiquitination by invading virus particles and degrade them or polyubiquitinate TNF receptor-associated factor (TRAF) 3 to activate the IFN response against the virus [23], while TRIM39 regulates the NF-κB signaling pathway to participate in immune defense [24]. Ftrs are teleost-specific TRIM-B30.2 genes, with no homologs identified in other vertebrates, while two evolutionarily close TRIM genes, TRIM16 and TRIM25, are found in a phylogenetic tree, which have also been reported to exert multiple antiviral functions [22]. These studies suggest specific selective pressure derived from virus–host interactions for the massive duplication of teleost TRIMs-B30.2 [68]. In the present study, we also identified seven hltrs, four btrs and one ftr in the grass carp genome, and we speculate that they might be involved in the immune defense against GCRV. We also analyzed the tandem repeat of grass carp TRIMs-B30.2 and tried to further reveal the mechanism for gene expansion, referring to previous hints from other teleost species [22]. Unexpectedly, no tandem repeat was found in the identified *Ci*TRIMs by MCScanX. We think that the fewer *Ci*TRIMs identified due to the low-quality assembly of the extant grass carp genome may partly explain this, because genome assembly with DNA fragment shifting or mutation can mask gene tandem repeats [47]. In addition, the MCScanX analysis of gene tandem repeats requires the genome to be assembled to the chromosome level [51], while the extant genome of grass carp in this study was only assembled to the draft level and with only 114 out of 301 scaffolds anchored on linkage groups, which may also have led to biased results for the gene tandem repeats. However, the real reason should be further explored in the future.

It has been reported, in teleosts, that WGCNA and gene expression trend analysis represent two effective methods for identifying anti-infection immune gene co-expression networks or temporal gene expression profiles and revealing their related immune processes based on transcriptome data [29,30]. For example, Ning et al. employed a gene expression trend analysis combined with WGCNA to identify the differentially expressed gene clusters associated with immune processes, including cytokine–cytokine receptor signaling, Toll-like receptor signaling and other immune-related pathways from the transcriptomes of Japanese flounder (*Paralichthys olivacrus*) infected with *Vibrio anguillarum* [33]. Given the importance of specific TRIMs in animal antiviral immunity [2,9], we reanalyzed two published grass carp transcriptomes during GCRV infection to seek potential antiviral *Ci*TRIMs based on their expression patterns. In the spleen transcriptomes after GCRV challenge at 1, 3, 5 and 7 days, we identified three significantly differentially expressed *Ci*TRIMs. A further WGCNA showed two out of these three *Ci*TRIMs, *Ci*btr40 and *Ci*TRIM103, which both contain the B30.2 domain, were clustered into the co-expression network that contains vital type I IFN response pathway genes such as RIG-I-like receptor, MDA5, STAT1, ISG58, PKR, viperin and MX1. The genes in this co-expression network have mainly been associated, by GO enrichment analysis, with immune processes including defense responses to other organisms, cellular responses to cytokine stimulation and cysteine-type endopeptidase activity involved in apoptosis. To find more potential antiviral *Ci*TRIMs, the CIK transcriptomes after GCRV challenge at 0 h, 6 h, 12 h and 24 h were also reanalyzed, and a total of eight significantly differentially expressed *Ci*TRIMs were identified. It has been reported that gene expression trend analysis is more suitable than WGCNA for the transciptome data with less than 15 samples [61]. A further gene expression trend analysis was conducted for the CIK transcriptomes and showed that one of these eight *Ci*TRIMs, *Ci*TRIM109, which is also a TRIM-B30.2, shows an expression trend significantly similar to that of the gene cluster that is enriched in the regulation of type I IFN production and the NOD-like receptor pathway. Expectedly, our qPCR results verify that 11 differentially expressed *Ci*TRIMs identified from the transcriptomes, including *Ci*btr40, *Ci*TRIM103 and *Ci*TRIM109, were significantly upregulated along with the upregulation of *Ci*IRF3, an important IFN regulatory factor [63], after GCRV challenge at 24 h. Increasing evidence has shown that certain TRIMs, especially TRIM-B30.2, can regulate RIG-I-like receptor, NOD-like receptor and the MDA5-mediated type I IFN response and promote the production of antiviral molecules, including ISGs, PKR, viperin and MX1, in various species [13,27]. We admit that the results for the expression profiles obtained by the qPCR analysis are not sufficient for the functional identification of actual antiviral TRIMs in grass carp. Nevertheless, our results strongly indicate that *Ci*btr40, *Ci*TRIM103 and *Ci*TRIM109, all containing the B30.2 domain, are associated with the type I IFN response during GCRV infection according to WGCNA and gene expression trend analysis, suggesting their involvement in the antiviral immunity of grass carp.

## 5. Conclusions

**Figure 8:**
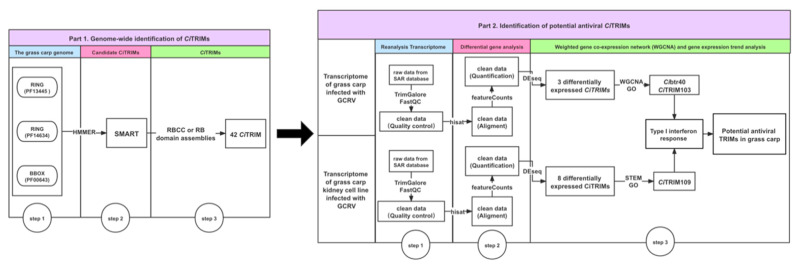
The overall framework for genome-wide identification of potential antiviral TRIMs in grass carp using transcriptomes in this study. First, the candidate *Ci*TRIMs containing RBCC, or RB domain assemblies were searched from the grass carp genome using the HMMER software, followed by removing redundancy through SMART analysis; finally, 42 *Ci*TRIMs were identified. Secondly, two published transcriptomic datasets based on experiments with grass carp individuals and cells infected with GCRV were downloaded from the SRA database (SRP095827, PRJNA597582 and PRJNA597542), followed by reanalysis. Eleven differentially expressed *Ci*TRIMs were identified through fold changes > 2 and FDRs (or adjusted *p*-values) < 0.05 using the DESeq R package. Through WGCNA and gene expression trend analysis, 3 TRIMs-B30.2, including *Ci*TRIM103, *Ci*btr40 and *Ci*TRIM109, associated with the type I interferon response during GCRV infection, were deduced as potential antiviral TRIMs in grass carp.

In conclusion, this study identified a total of 42 *Ci*TRIMs from the grass carp genome, which possessed conserved RBCC or RB domain assemblies at their N-termini and eight different domains at their C-termini. Among them, 19 *Ci*TRIMs contained the B30.2 domain at their C-termini, which has previously been proved to be fast-evolving and to play important roles in the antiviral immune defense. We also found a total of 11 significantly differentially expressed *Ci*TRIMs in two transcriptomes during GCRV infection. Despite the lack of further functional verification, three of them, including *Ci*btr40, *Ci*TRIM103 and *Ci*TRIM109, all belonging to TRIMs-B30.2, are associated with the type I IFN response during GCRV infection and deduced as potential antiviral TRIMs in grass carp (Figure 8). These findings may offer useful information for understanding the structure, evolution, and function of TRIMs in teleosts and provide potential antiviral immune molecule markers for the disease resistance breeding of grass carp.

## Figures and Tables

**Table 1:** The qPCR primers used in this study.

Primer Name	Primer Sequence (5′–3′)
*Ci*TRIM2-F	TGGTGCGTCAGATCGACAAA
*Ci*TRIM2-R	CTGTGGGCGGGAATGTAGTT
*Ci*TRIM35-16-F	TCTGGTTCCTGTCCTCAATGC
*Ci*TRIM35-16-R	TGTTAGCCACAATGCGGTTG
*Ci*TRIM-35-50-F	CCTCCAGTCAATCAGGCTCT
*Ci*TRIM-35-50-F	ATTTCCTTTGTTGCCTCTGCT
*Ci*btr40-F	AAAAGACAGCAGTGCAGCAG
*Ci*btr40-R	CGATCTCCTTCTCTTTGGCTTG
*Ci*TRIM46b-F	TAGAAAGCGGCATTGCTCAG
*Ci*TRIM46-R	ACCACGCAATTCACTCACAC
*Ci*TRIM5-like-F	ACGCCATTGATGCTCTTGTG
*Ci*TRIM54-like-R	TTGGCACGTTGAGCATTGTC
*Ci*TRIM71-F	ACCATCGCATTCAGGTGTTCG
*Ci*TRIM71-R	TCATTCCATCTGGGGTAACCGCTA
*Ci*TRIM103-F	CCACCTTCATTGCCCCATCT
*Ci*TRIM103-R	GCGTCTGGTAAAATTCCCGC
*Ci*TRIM109-F	AACAGATCCAGTGCTCCGTG
*Ci*TRIM109-R	CTGCATTCCGGACACAGTCT
*Ci*TRIM110-F	TGCACAATTTCAGCACCAGC
*Ci*TRIM110-R	GATGGTGACCCTGCTGTTCA
*Ci*TRIM112-F	TCCAGAACCACCCGCTTGTGA
*Ci*TRIM112-R	CCCCTTGTGCGACCCAACCAG
IRF3-F	ACTTCAGCAGTTTAGCATTCCC
IRF3-R	GCAGCATCGTTCTTGTTGTCA
VP2-F	ATCAAGGATCCCATTCCGCCTTCA
VP2-R	TTAGAGGATCGTGCCATTGAGGGT
β-actin-F	GCTATGTGGCTCTTGACTTCG
β-actin-R	GGGCACCTGAACCTCTCATT

Note: F, forward primer; R, reverse primer.

**Table 2:** Overall information for the 42 *Ci*TRIMs identified in this study.

Gene Name	Genome ID	PL (aa)	MW (KDa)	PI	EN	PSL
*Ci*TRIM1	CI01000000_14975127_14983931	404	45.89	8.63	4	cytoplasm
*Ci*TRIM2	CI01000300_10176172_10189470	812	89.11	6.20	12	cytoplasm
*Ci*TRIM3	CI01000304_12076818_12085420	784	86.13	8.11	12	cytoplasm
*Ci*TRIM3a	CI01000095_00776224_00788366	770	84.11	7.53	13	cytoplasm
*Ci*TRIM13	CI01000009_00343750_00344964	404	45.63	5.92	1	cytoplasm, nucleus
*Ci*TRIM18	CI01000349_00034813_00052670	676	75.74	6.32	9	cytoplasm, cytoskeleton
*Ci*TRIM23	CI01000304_04650911_04659754	579	64.74	6.03	11	cytoplasm, nucleus
*Ci*TRIM25	CI01000112_00810157_00821637	473	53.60	8.65	5	cytoplasm
*Ci*TRIM25-like	CI01000354_01204555_01213381	405	46.89	6.65	6	nucleus
*Ci*TRIM32	CI01000059_09706218_09708197	659	72.50	6.58	1	nucleus
*Ci*TRIM33-like	CI01000027_07545610_07559939	1326	14.64	8.00	19	nucleus
*Ci*TRIM35-1	CI01000258_00137907_00154325	525	59.04	8.27	8	nucleus
*Ci*TRIM35-13	CI01000113_01547153_01553082	387	44.07	8.25	5	cytoplasm
*Ci*TRIM35-16	CI01000158_00190147_00195261	401	45.70	8.51	6	cytoplasm, nucleus
*Ci*TRIM35-17	CI01000087_02092947_02096033	544	61.76	6.31	6	cytoplasm
*Ci*TRIM35-29	CI01000027_06329025_06333271	406	46.45	8.43	6	cytoplasm, nucleus
*Ci*TRIM35-30	CI01000013_11271292_11274155	271	31.64	8.80	3	nucleus
*Ci*TRIM35-50	CI01000013_03911586_03916513	411	47.45	8.29	6	cytoplasm, nucleus
*Ci*TRIM39-like	CI01000196_00233827_00236332	541	59.59	6.39	2	cytoplasm
*Ci*TRIM45	CI01000009_10250936_10259244	568	62.21	7.97	7	cytoplasm
*Ci*TRIM46b	CI01000027_04750483_04762663	753	83.61	7.65	11	cytoplasm, nucleus
*Ci*TRIM47	CI01000304_12065676_12071391	491	56.31	5.89	8	cytoplasm, nucleus
*Ci*TRIM54	CI01000029_01758347_01773935	380	43.11	5.18	9	cytoskeleton
*Ci*TRIM54-like	CI01000009_08283637_08293491	575	64.21	4.93	7	nucleus
*Ci*TRIM55a	CI01000098_02956318_02961605	419	47.23	4.95	9	cytoplasm, nucleus
*Ci*TRIM55b	CI01000018_06417005_06423657	379	43.18	5.06	8	cytoplasm, cytoskeleton, nucleus
*Ci*TRIM59	CI01000092_04869145_04870424	425	47.95	6.03	1	cytoplasm
*Ci*TRIM63	CI01000024_00581310_00583174	371	41.93	5.34	2	cytoplasm, nucleus
*Ci*TRIM67	CI01000051_06837978_06881158	713	79.43	6.58	12	cytoplasm, cytoskeleton
*Ci*TRIM71	CI01000016_10600154_10633808	934	10.27	6.61	5	cytoplasm
*Ci*TRIM101	CI01000001_04746153_04754209	465	52.81	4.87	10	cytoplasm, nucleus
*Ci*TRIM103	CI01000354_01417265_01426361	491	55.39	6.01	4	cytoplasm, nucleus
*Ci*TRIM109	CI01000016_05986400_05994821	501	56.96	6.29	7	cytoplasm, nucleus
*Ci*TRIM110	CI01000004_15999644_16005705	469	53.57	6.58	7	cytoplasm, extracellular
*Ci*TRIM111	CI01000012_13551086_13554035	545	62.27	5.77	6	cytoplasm
*Ci*TRIM112	CI01000180_01434810_01440307	493	54.31	5.98	4	nucleus
*Ci*RNF207	CI01000001_06965170_06976281	633	72.34	5.96	17	nucleus
*Ci*btr1	CI01000119_00092461_00099753	592	66.61	7.82	6	cytoplasm
*Ci*btr11	CI01000344_01402598_01409449	468	52.78	6.17	6	cytoplasm, nucleus
*Ci*btr12	CI01000354_01450329_01466394	645	73.13	8.73	8	cytoplasm
*Ci*btr40	CI01000344_01289427_01295915	520	60.11	6.65	5	cytoplasm
*Ci*ftr83-like	CI01000339_06060658_06063401	299	34.30	8.21	4	cytoplasm

Note: PL, protein length; MW, molecular weight; PI, isoelectric point; EN, exon numbers; PSL, predicted subcellular localization.

## Data Availability

The grass carp genome and tissue expression data were downloaded from the Grass Carp Genome Database (GCGD, http://bioinfo.ihb.ac.cn/gcgd/php/index.php, accessed on 20 November 2020) [39]. The transcriptome raw data (accession number: SRP095827) [53] for the spleens from grass carp infected with GCRV and the raw transcriptome data (accession numbers: PRJNA597582 and PRJNA597542) [60] for the grass carp kidney cell line (CIK) after GCRV challenge are available in the public NCBI database.
